# Midwives’ and patients’ perspectives on disrespect and abuse during labor and delivery care in Ethiopia: a qualitative study

**DOI:** 10.1186/s12884-017-1442-1

**Published:** 2017-08-22

**Authors:** Sahai Burrowes, Sarah Jane Holcombe, Dube Jara, Danielle Carter, Katheryn Smith

**Affiliations:** 1 Public Health Program, College of Education and Health Sciences, Touro University California, 1310 Club Dr. Mare Island, Vallejo, CA 94592 USA; 2 Bixby Center for Population, Health, and Sustainability, University of California, Berkeley 17 University Hall, Berkeley, CA 94720-7360 USA; 3 Department of Public Health College of Medicine and Health Sciences, Debre Markos University, Debre Markos, Ethiopia; 4 Touro University California, 1310 Club Dr. Mare Island, Vallejo, CA 94592 USA

**Keywords:** Midwives, Respectful maternity care, Disrespect and abuse, Maternity care, Quality, Patients’ rights, Woman-centred care

## Abstract

**Background:**

It is increasingly recognized that disrespect and abuse of women during labor and delivery is a violation of a woman’s rights and a deterrent to the use of life-saving, facility-based labor and delivery services. In Ethiopia, rates of skilled birth attendance are still only 28% despite a recent dramatic national scale up in the numbers of trained providers and facilities. Concerns have been raised that womens’ perceptions of poor quality of care and fear of mistreatment might contribute to this low utilization. This study examines the experiences of disrespect and abuse in maternal care from the perspectives of both providers and patients.

**Methods:**

We conducted 45 in-depth interviews at four health facilities in Debre Markos, Ethiopia with midwives, midwifery students, and women who had given birth within the past year. Students and providers also took a brief quantitative survey on patients’ rights during labor and delivery and responded to clinical scenarios regarding the provision of stigmatized reproductive health services.

**Results:**

We find that both health care providers and patients report frequent physical and verbal abuse as well as non-consented care during labor and delivery. Providers report that most abuse is unintended and results from weaknesses in the health system or from medical necessity. We uncovered no evidence of more systematic types of abuse involving detention of patients, bribery, abandonment or ongoing discrimination against particular ethnic groups. Although health care providers showed good basic knowledge of confidentiality, privacy, and consent, training on the principles of responsive and respectful care, and on counseling, is largely absent. Providers indicated that they would welcome related practical instruction. Patient responses suggest that women are aware that their rights are being violated and avoid facilities with reputations for poor care.

**Conclusions:**

Our results suggest that training on respectful care, offered in the professional ethics modules of the national midwifery curriculum, should be strengthened to include greater focus on counseling skills and rapport-building. Our findings also indicate that addressing structural issues around provider workload should complement all interventions to improve midwives’ interpersonal interactions with women if Ethiopia is to increase provision of respectful, patient-centered maternity care.

**Electronic supplementary material:**

The online version of this article (doi:10.1186/s12884-017-1442-1) contains supplementary material, which is available to authorized users.

## Background

This study examines the experience of disrespect and abuse during labor and delivery in Ethiopia through the juxtaposition of in-depth interviews with midwives, midwifery students, and women who have recently given birth. Disrespect and abuse of women during labor and delivery has become an increasingly recognized phenomenon over the past decade. Global public health norms now explicitly condemn such practices, acknowledging them as both a violation of a woman’s rights and also, instrumentally, as a deterrent to the use of life-saving facility-based labor and delivery services [1–3] .

Low levels of facility-based delivery are one of the drivers of maternal deaths, and delivery with a skilled birth attendant can significantly reduce maternal mortality [4–6]. While multiple factors explain low health service utilization, there is increasing recognition that many women are reluctant to use reproductive, maternal, neonatal, and child health (RMNCH) services because of poor service quality and fears of provider mistreatment. Numerous studies demonstrate that women’s perceptions of how they will be treated at health facilities can strongly influence their choice about where to deliver, and deter women from accessing services in a timely manner, or at all [7, 8]. Unfortunately, disrespect and abuse of patients, particularly during childbirth, persists globally and is prevalent throughout East Africa [7, 9–15].

Despite the Ethiopian Ministry of Health’s prioritization and vigorous support of efforts to reduce maternal and child mortality [2], underutilization of RMNCH services remains a problem in Ethiopia and contributes to the country’s high maternal mortality rates: 420 women die for every 100,000 live births in the country and maternal deaths constitute 21% of all deaths to women ages 15–49 [16]. Currently, only 28% of women receive skilled health care services at delivery [17]. This low utilization in Ethiopia has been shown to be associated with women’s education levels, residence, ethnicity, parity, autonomy and household wealth, among other factors [18–20]. Studies in Ethiopia also show that perceptions of poor quality of care such as lack of privacy and lack of psychosocial support, are significant factors in a woman deciding whether or not to give birth at a health facility [21, 22]. Furthermore, recent studies reveal evidence of disrespect and abuse in Ethiopian facilities [15, 23–25]. For example, findings from women and providers in health facilities in two regions found that 21% of post-partum women surveyed reported disrespect and abuse, non-consented care (17.7%), lack of privacy (15.2%), and non-confidential care; and 82% of providers cited occurrences of disrespect and abuse in their facilities [26]. Nonetheless, Ethiopia has enshrined promotion of women’s rights and status in its constitution and subsequent national policies [27], and has supported the core United Nations General Assembly resolutions and other international agreements that acknowledge the rights of childbearing women to respectful maternity care [1, 26–33]. However, individual patients are unlikely to know about, much less use, any mechanisms to address rights violations.

In an effort to reduce maternal mortality, Ethiopia’s government has both expanded health care infrastructure and coverage and has undertaken initiatives to make care more hospitable. These include expanding numbers of midwives trained and posted in rural areas (matched with their region of origin); operationalizing a Women’s Health Development “Army” to conduct health outreach to rural women, and providing traditional foods to women who give birth in rural health centers [34, 35]. A distinctive feature of the expansion of the midwifery profession is the growing proportion of male midwives (22%) due to new, exam-based selection criteria [36].^1^ Relatively understudied challenges of this scale-up are ensuring the quality of these services and understanding women’s readiness to use them. This study aims to help address this knowledge gap.

### Theoretical framework

Mistreatment of women during labor and delivery has persisted across time and geography and has been given numerous names including “obstetric violence” and “dehumanized care”. There is growing global commitment to addressing this challenge, which has been buttressed by policy statements from the World Health Organization [2], the Lancet [42] and notably the White Ribbon Alliance’s 2011 facilitation of the Respectful Maternal Care Charter [1], a global consensus statement on a positive vision for respectful maternity care with a definition of disrespect and abuse and the corresponding rights (see Table 1 below). This Charter has been translated into eight languages and shared among providers, health managers, and advocates [3], and is anchored in United Nations and other international commitments signed by most national governments.

**Table 1:** Respectful Maternity Care: Charter on the Universal Rights of Childbearing Women

Tackling Disrespect and Abuse: Seven Rights of Childbearing Women	
Category of disrespect and abuse	Corresponding right
Physical abuse	Freedom from harm and ill treatment
Non-consented care	Right to information, informed consent and refusal, and respect for choices and preferences, including companionship during maternity care
Non-confidential care	Confidentiality, privacy
Non-dignified care (including verbal abuse)	Dignity, respect
Discrimination based on specific attributes	Equality, freedom from discrimination, equitable care
Abandonment or denial of care	Right to timely healthcare and to the highest attainable level of health
Detention in facilities	Liberty, autonomy, self-determination, and freedom from coercion

*White Ribbon Alliance, 2011*

Systematic reviews of studies of disrespect and abuse during labor and delivery highlight both structural and individual drivers. They find that abuse is not limited to a few individuals or institutions, but rather is reflective both of systemic failures as well as of deeply embedded provider attitudes and beliefs [10, 11, 14, 43–45]. Structural factors identified include provider shortages/heavy workloads, poor physical infrastructure, lack of supplies and equipment, and lack of supervision [46]. Such conditions are particularly prevalent in sub-Saharan Africa, and are often associated with individual-level drivers of abuse such as provider stress, overwork, low motivation and stigmatizing attitudes [10, 14, 47]. General lack of supportive care and poor communication between providers and patients are also often considered forms of disrespect and abuse. Systematic reviews show that patients view both intentional and unintentional mistreatment as abusive [10].

Our study makes two contributions to this emergent literature. It is one of the few to triangulate findings and contrast provider and patient perspectives by studying patients as well as both practicing and student providers. It also offers a new examination of the relationship between disrespect and abuse, providers’ knowledge of patients’ rights, and their behavior in clinical scenarios.

## Methods

The overall goal of this cross-sectional, qualitative study was to examine the nature of disrespect and abuse in midwifery care during labor and delivery in the Debre Markos area. The specific research aims were to:

1. Examine women’s experiences of care from midwives during labor and delivery, including any disrespect or abuse;
2. Explore midwives’ understandings of patients’ rights and patient-centered care;
3. Describe midwives’ experiences of patient abuse and disrespect;
4. Identify patient and midwife recommendations for strengthening the quality of labor and delivery care.

### Setting

This project took place in Debre Markos, a city in Amhara region, located 5 h northwest of Ethiopia’s capital. A joint team of researchers from Debre Markos University’s (DMU) Department of Public Health, Touro University California’s Public Health program and the Bixby Center for Population, Health, and Sustainability, at the University of California, Berkeley, conducted this research. Data collection took place on the DMU campus in the School of Midwifery, at Debre Markos three main public health centers (the Hidase, Gozeman, and Debre Markos Health Centers), and at the Debre Markos Referral Hospital in February and March, 2015.

### Sample

The study examined two populations: women who had recently given birth and midwifery professionals (both students who were providing care and practicing midwives). A convenience sample of 23 women over the age of 20 who had given birth attended by a midwife within the past year, was recruited from health facilities to take part in an open-ended interview. Three women who had given birth at home were recruited by Health Extension Workers and interviewed. The study also conducted in-depth interviews with fifteen randomly selected (93% response rate) third-year bachelor’s degree midwifery students from DMU and four purposively sampled practicing midwives from the study health facilities. Patients were recruited as they left the postnatal or well-baby clinics at Debre Markos health centers or the Debre Markos referral hospital on a first-come first-served basis.

Both the provider and patient samples were stratified by the gender of the midwife provider. Midwifery students were drawn from gender-separated, numbered class lists and were randomly selected using SPSS’s random number generator. Recruiters selected two males and two female practicing midwives from the study health centers and screened patients during recruitment to ensure that at least one-third of the patients had had male midwives at their last delivery.

### Interview guide

Interview guides and surveys (Additional file 1) were developed in English, reviewed with colleagues in the DMU Department of Public Health, and then translated into Amharic by a professional translator of reproductive health materials. The instruments were pre-tested for length and comprehensibility with a sample of five women at the Debre Markos Health Center and five midwifery students at the DMU School of Midwifery. A project investigator debriefed interviewers and the project coordinators after pre-testing and confirmed the faithfulness of the survey and transcript translation.

All interview guides contained questions on respondent demographics and socio-economic characteristics. To address our first research aim, patients were asked about the quality and content of their care during pregnancy and labor and delivery, the quality of their interactions with healthcare providers, their satisfaction with the care they received, and about their knowledge of the quality of other women's experiences during labor and delivery. They were also asked directly about common forms of mistreatment that might not be perceived as abuse, such as the denial of food and drink during labor, refusal of accompaniment, and not being able to give birth in their desired position.

In order to gather provider perspectives on disrespect and abuse of patients (research aim two), we asked midwives and midwifery students about their experiences of provider-patient interactions, and their observation and/or awareness of patient mistreatment. Our third research aim was to examine provider’s understanding of patients’ rights in order to see whether gaps in their knowledge could contribute to patient disrespect and abuse. Midwifery students and midwives were, therefore, also asked questions on the coverage of patients’ rights in midwifery training. In addition, they also responded to a short self-administered survey on their knowledge of patients’ rights.

To enable us to contrast midwives’ understanding of patients’ rights with the degree to which they might observe these rights in practice, we questioned them about their knowledge and comfort with service provision in two clinical scenarios where official policy, correct medical practice, and respectful medical care likely conflict with prevailing cultural beliefs, leading to poor quality of care. One scenario gauged their willingness to provide contraception to an unmarried adolescent, the other, their comfort with providing abortion care services. Although the Ethiopian government has actively promoted access to contraception and has liberalized its laws on abortion [48], Ethiopia remains culturally conservative with 67% of the population regarding abortion as “never justifiable” [49] and premarital intercourse for women relatively rare and culturally discouraged [50].

To address our fourth research aim, both patients and providers were asked about the reasons they thought abuse occurred and their recommendations for improving the quality of labour and delivery care.

### Data collection

The study used four masters-level interviewers, recruited from the DMU Department of Public Health who had carried out survey- or interview-based data collection previously. We chose interviewers who were outside the departments that train health professionals (midwives, pharmacists, physicians) in order to reduce age and power differentials with study participants, as well the chances of interviewees knowing interviewers.

The interviewers participated in a three-day workshop covering the motivation for the study, a refresher on research ethics, project data collection and management procedures, an overview of qualitative methods, and practice using interviewing techniques.

Patient interviews took place at coffee stands near to the health facility or in private rooms in the health facility, depending on availability and the patients’s choice. For the three patients who gave birth at home, interviews were conducted in their homes. Student interviews took place in private rooms on the Debre Markos campus.

All interviews were conducted in Amharic, audio-recorded, and then simultaneously translated and transcribed into English by a single professional translator conversant with the reproductive health field. The project coordinator held weekly debriefing sessions with interviewers to discuss experiences and surprises encountered during the interviews and to refine the interview guide. In addition, project investigators reviewed interview debrief memos and interview transcripts as they were translated, and provided ongoing feedback and suggestions for making the interviews more open and consistent.

We developed a codebook (Additional file 2) with a priori codes guided by the framework of Bowser and Hill, the categories of disrespect and abuse and rights defined in the Charter of Respectful Maternity Care [1, 14], and review of the disrespect and abuse literature. We performed deductive and inductive thematic content analysis of interview transcripts: using a priori codes for initial rounds of analysis and adding new codes to reflect themes emerging from the data.

Coding was conducted separately for the two population groups. One investigator was responsible for coding responses of midwives and students, another for patient responses. After completing coding for providers and patients, coders shared results and noted common themes and divergences both within and between the samples. This approach, keeping the samples separate, may have limited the tendency for coders to expect, and therefore find, codes in their sample based on the responses found in the other study groups. It did, however, prevent us from conducting tests of inter-rater reliability. The coding and analysis was conducted using the HyperResearch version 3.73 qualitative data analysis software.

## Results

### Sample characteristics

Our patient sample had more educated and older first-time mothers than is the case nationally in Ethiopia. Nationally, age at first birth among those ages 25–29 is 19.6, and 48% of Ethiopian women have no education, and only 12% and 6% have some secondary education or some tertiary education, respectively [17]. In this study, over a quarter of women delivering had some secondary education and almost a quarter had some tertiary education and a few had completed master’s degrees. Our sample of midwifery professionals had a slightly higher proportion of male midwives than the current national average, and over half of the women delivering in our sample were served by a male midwife, but the provider sample was otherwise comparable to national averages (see Table 2 below) [36, 51].

**Table 2:** Sample characteristics

	Patients		Students & providers	
	n	%	n	%
Sex of midwife (female)^a^	11	48%	13	68%
Age group				
*18–20*	1	3%	5	26%
*21–25*	17	65%	12	63%
*26–30*	5	19%	2	11%
*31 or older*	3	12%	0	0%
Number of previous children^b^				
*>3 children*	2	8%	0	0%
*1–3 children*	8	31%	1	5%
*0 children*	16	62%	18	95%
Level of Education	*Own education*		*Father’s education*	
*None*	7	30%	13	72%
*Some Primary*	5	22%	1	6%
*Some Secondary*	6	26%	2	11%
*Some Tertiary or higher*	5	22%	2	11%
Marital status (single)	0	0%	19	95%

Notes: *n* = 45 (26 women; 15 midwifery students; 4 practicing midwives)

^a^Only patients who gave birth at a healthcare facility were asked about the attending midwife’s gender

^b^Among midwives, only married interviewees were asked how many children they had

### Research aim 1: patient experiences of care

Our first research aim was to examine women’s experience of labor and delivery care including instances of disrespect and abuse. We started interviews by asking women to describe their experiences of care and their perceptions of its quality. Most of the patients interviewed reported satisfaction with their care during labor and delivery. Women who experienced disrespect and abuse during labor and delivery also reported lower satifaction with care. Patients were aware that health facilities offer life-saving care and seemed grateful for access to care, particularly because services were offered free of charge and included interventions such as drugs to prevent postpartum hemorrhage and vertical transmission of HIV, which they knew could not be easily provided in a home birth.

*...the blood loss here is minimal. Prevention of mother-to-child transmission of HIV is well done here...They take maximum care when they cut the umbilical cord which helps prevent vertical transmission of HIV. They also give injections to control blood loss. (Patient #21)*

*Had the provider not given me the care, I wouldn’t have been saved. It was an operative* [caesarian] *delivery. (Patient #40)*

Patient interview responses indicate that women ‘shopped’ for care, going out of their way to attend facilities with a reputation for providing quality care. The quality of care offered at the facility also seemed to be a factor in women's choice of home rather than facility birth.

*Their care here is very good. My friend delivered here 3 years ago and she advised me to deliver here. In fact this is a far place* [from home]. *(Patient #21)*

Patients’ responses revealed some indications that Health Extension Workers may play a role in alerting women to quality problems at particular facilities and encouraging women to attend facilities with reputations for more respectful care.

*... a lady who gave birth at the hospital was mistreated; providers were shouting at her and left her while she was in pain...after that, the health extension workers taught us in the villages and I came here* [to the health center] *for care….After being informed by the health extension workers, I came here and I found them very comforting; otherwise, Gozamen* [health center] *was nearer to me than this health center. (Patient #22)*

There was no evidence that patients with male midwives had more negative labor and delivery experiences than those with female midwives and patients reported high acceptability of male midwives. The majority of women interviewed either expressed no gender preference or said that they preferred male midwives, citing their greater professionalism, empathy, and competence.

*The female* [midwives] *do not give you freedom in positioning. The male* [midwives] *allow free movement… as well as walking. The females also insult and shout at patients. So I prefer the males. (Patient #32)*

### Research aims 1 and 3: observation and experience of disrespect and abuse

Both midwives and patients reported having observed or heard of disrespect and abuse of patients during labor and delivery, however, the types of abuses reported varied markedly between the two groups. In response to direct questions about personal experiences of disrespect and abuse, both patients and providers first, and most frequently, mentioned verbal abuse. However, after combining answers to direct questions about abuse with *indirect* mentions of abuse that emerge from answers to questions on observations of care, ways to improve service quality, and personal experiences during labor and delivery, a discrepancy emerges. Patients are most likely to mention denial of preferred birth position (which they do not always identify as a form of abuse), while providers report verbal abuse as the leading type of violation (see Table 3).^2^

**Table 3:** Patient and provider frequency rankings of observed or experienced disrespect and abuse by category

Category of disrespect and abuse	Patient rank	Provider rank
Non-consented care	(1) Denial of preferred birth position	
	(2) Denial of accompaniment	
Abandonment or denial of care	(3) Poor clinical practice, neglect	(4) Denial of services
Non-dignified care (including verbal abuse)	(4) Verbal abuse	(1) Verbal abuse
Physical abuse		(2) Physical abuse *(hitting, pinching, slapping)*
Non-confidential care		(3) Violation of privacy
Discrimination based on specific attributes	None reported	
Detention in facilities	None reported	

Top four mentions listed. Frequency rank in parentheses

#### Verbal abuse

Verbal abuse was reported by almost half of both patients (48%) and of midwives and midwifery students (43%). Patients reported that providers often shouted at them or at other patients, mocked them, or spoke to them in harsh tones.

*Since I was in pain, I told her* [the midwife] *to save me. She shouted at me and then I didn’t talk to her even when I was in pain. I refused to comply with her orders after that. When she asked me to push more, I didn’t assist her. (Patient #33)*

*Some of them heap scorn on you when you are in labor. (Patient #41)*

#### Physical abuse

We find a significant discrepancy in reports of physical abuse between patients and providers. None of the patients interviewed mentioned being physically abused themselves, although several mentioned hearing reports of abuse or witnessing physical abuse of other patients. In contrast, approximately a third of providers indicated they had personally observed physical abuse of patients. The most common type of physical abuse witnessed was slapping patients on the legs in order to get them to comply with midwives’ instructions for vaginal exams or for positioning for labor.

*Yes. I heard that they were pinching and slapping a client to open up her legs. (Patient #39)*

#### Patient autonomy

Most patients were allowed to drink liquids during labor, but food was frequently denied. Most patients were not allowed to give birth in their desired position, and a large minority were not permitted to have family members or friends accompany them during delivery. Patients usually did not report this lack of autonomy as abuse. They frequently deferred to the wisdom of providers with regard to birth positioning decisions, and many said that they did not want accompaniment during labor because they were concerned about privacy or because they did not want to distress family or worry about comforting others while in pain.

*Providers chose the appropriate position in a way not to distress my baby. (Patient #36)*

*No I didn’t* [want accompaniment], *because they would only suffer with me.... Since they were suffering with me, the providers told them to go out. (Patient #22)*

#### Non-consented care, abandonment, and poor clinical practice

Abuse often manifested in providers offering substandard or otherwise medically inappropriate care, sometimes without consent. Both patients and providers report witnessing and/or experiencing sub-standard clinical practice during labor and delivery. Midwives and midwifery students mentioned observing practices such as stitching episiotomies without anesthesia, performing procedures without informing the patient, and denial of follow-up care to patients who had previously refused services.

*I have seen verbal abuse and stitching episiotomy without anesthesia; but as to the latter, providers claim that using anesthesia may sometimes delay wound healing. I also have noticed procedures like episiotomy being conducted without informing the client. (Midwifery Student #2)*

Patient reports of poor clinical care include having to endure unnecessary procedures such as episiotomies and frequent vaginal examinations, having poor follow up care, and being given improper medication.

*What I disliked is the frequent vaginal examination they conducted. That could create edema* [swelling of the genitals]*, this is not good; second they get bored at times and maltreat clients. They also forget procedures sometimes; they didn’t order antibiotics for me when I was discharged, and I took it by myself. Sometimes, they don’t change beds as well. (Patient #38)*

In addition to disrespect and abuse in direct interactions with midwives, patients also frequently mentioned abandonment and neglect, and mistreatment due to weakness of the health system. Examples included rushed care, unsanitary rooms, crowding, and long periods of either waiting or of being left alone.

*There is a shortage of beds. No waiting area, we just roam around the facility. (Patient #32)*

*I went in the evening and met a lady midwife who evaluated me only once overnight and she didn’t reappear. I delivered in the morning and was assisted by the morning provider. She was called after part of the baby was delivered by itself; she didn’t care for me much. (Patient #32)*

#### Lack of privacy and confidentiality

Patients complained frequently about the lack of privacy on the wards due to the lack of screens or curtains and also due to the large number of students who observe deliveries as part of their training.

*I told the midwife not to allow* [students to enter and observe care]*, but they were already in the room on practical learning, and the midwife didn’t want to send them out once they were in. In the future, I don’t want that. (Patient #31)*

Some providers were acutely aware that having many students present at once on the wards compromised women’s privacy and their right to consent, and resulted in poor care:

*It* [verbal abuse] *happened in Debre Markos Hospital. We students were many in number, and clients got ashamed to be free in front of us* [to permit vaginal exams]*, and did not comply with the orders given by providers; this is worsened by the large number of delivering mothers served in the hospital. Providers will be in a rush and will shout at the clients. (Midwifery Student #11)*

### Research aim 2: provider knowledge of patients’ rights

We find that providers currently receive limited training on patients’ rights; and when discussing training, referred to professional ethics rather than patients’ rights. Respondents report that the professional ethics content in the midwifery curriculum focuses primarily more narrowly on protecting patient confidentiality and privacy.

*Yes we have taken* [training] *to keep patient privacy. That means to keep any secret about the patient that has a potential to be irritating to her. (Midwifery Student #2)*

*I didn’t take any* [special patient’s rights training]. *But I remember some part of it: if a mother comes to the facility, the midwife has the obligation to protect her from any infection. She has the right to get the service she came for. She has the right that her secrets be kept confidential. She has the right for her questions have to be answered. (Midwifery Student #7)*

Despite this lack of training, providers were uniformly positive in acknowledging the importance of patients’ rights, and most were able to correctly identify several key rights of patients receiving care during labor and delivery. Approximately half of practicing midwives and two-thirds of students reported that their training covered at least one of these rights. Further, the awareness of both patients and the providers of many of the behaviors that constitute rights violations was high. However, a wide variation in belief about whether women should be able to choose their birthing position, as suggested by right to respect for patient choices and preferences in the Respectful Maternity Care Charter [1], was an exception to this finding, and seems to reflect training on using certain positions to reduce particular health risks.

*Yes, mainly patient privacy is the primary one* [rule]*. The other is positioning during labor and delivery; it is advised to position her on left lateral to prevent hypotension. (Midwifery Student #5)*

In open-ended questions, when asked to define patients’ rights, providers and students described it, in order of frequency, as providing services and good treatment, protecting confidentiality, giving the patient a choice of provider, ensuring consent to procedures, proving an explanation of procedures to patients, respecting patient privacy, and giving choice of birth position.

Most student and practicing providers saw respecting patients’ rights as a fundamental factor in developing a positive relationship between patients and providers. However, they tended to view respect for patients’ rights instrumentally, as a mechanism for increasing skilled birth attendance, rather than a goal in itself.

### Directive counseling, stigma, and quality of care

A key aspect of providing patient-centered care involves counseling patients in a manner that promotes informed choice and shared decision-making. This is in contrast to directive counseling, or even outright commands, where providers aggressively promote specific health behaviors and leave little room for patient involvement in decision-making [52]. Respectful patient-centered care also requires health professionals to provide respectful care even when patients request a service or have a condition that is socially stigmatized. While stigmatization and directive counseling are not commonly thought of as forms of mistreatment in themselves, they can be considered indicators of provider respect for patients’ rights to information, informed consent and refusal, and timely care [1]. To examine these indicators, we asked midwives and midwifery students about whether, and in what manner, they would provide medically appropriate care for two socially stigmatized services. Their responses revealed deviations from respectful care (please see Table 4).

**Table 4:** Midwives’ respect for patient rights in clinical care scenarios (*self-report*)

Category of disrespect and abuse	Self-reported midwife behavior	Contraception for unmarried, unaccompanied adolescent	First trimester abortion care services for married mother of two
		*Scenario 1*	*Scenario 2*
Abandonment or denial of care	Was not willing to provide care	16%	32%
Non-dignified care	Displayed stigmatizing attitudes	0%	32%
Non-consented care; non-confidential care	Used directive counseling or unnecessary procedures	47%	58%

*n = 19 (15 midwifery students, 4 practicing midwives)*

When asked about what actions they would take when serving an unmarried adolescent who requested contraception and confidentiality about her request (which is in accordance with official policy in Ethiopia), most health providers said that they were willing to offer care. None used terminology or described actions that were stigmatizing. However, almost half said that they would counsel the patient in a directive manner.

*I will dig out why she wanted this to be kept as secret. Then I will choose what is appropriate for her and counsel...I will choose what is good for her and convince her. (Midwifery Student #1)*

In contrast to the relatively high willingness to provide contraception to unmarried adolescents, respondents struggled with their decisions about whether to provide abortion care services. Forty percent of respondents said that they would not be willing to provide abortion services given the scenario. In their descriptions of how they would care for hypothetical patients, a third displayed stigmatizing attitudes toward the patient, and two-thirds said that they would undertake some form of directive counseling or require the patient to undergo unnecessary checks and procedures.

*Even the Almighty doesn’t like this. I will advise her to have good antenatal care and use contraception after delivery. (Midwife #14)*

*I will counsel her to bear this baby and to take contraceptives afterwards. (Midwife #18)*

*I know the* [legal] *conditions of abortion and I can decide. (Midwife #7)*

### Research aim 4: recommendations for change

Our fourth research aim was to identify patient and midwife recommendations for strengthening the quality of labor and delivery care provided by midwives. We began our exploration of this topic by asking providers and patients why they thought abuse occurred.

#### Explanations for abuse

Providers described abuse as being the unintentional result of overwork and tiredness caused by very high patient loads and stress from having to negotiate critical, life-threatening interactions.

*But all* [of the abuse] *did not happen on purpose. When the baby is at risk of suffocation and the mother needs comfort, sometimes the providers verbally abuse them for their own sake. Sometimes they slap the thigh of the client. (Midwife #4)*

*Sometimes when there is a work overload, I may not strictly adhere to the professional ethics and rights I described to you earlier. (Midwifery Student #19)*

Even when they were specifically asked about what, beyond infrastructure shortcomings, they saw as the causes of disrespect and abuse, a substantial majority of midwives continued to emphasize structural factors (shortages of personnel, lack of supplies, heavy workload, and midwife fatigue) as causes of poor quality care and/or mistreatment. However, some acknowledged that even in high-stress, overburdened situations, not all midwives abused their patients.

Patients also saw provider stress and workload as the primary drivers of abuse but advised that providers should heed their professional and ethical commitments and find ways to better manage responsibilities and stress.

*Sometimes providers get upset for no reason, maybe because they are tired (Patient #24).*

*If there is work overload, they had better share responsibility to avoid burn out. (Patient #31)*

Low remuneration and boredom were also mentioned by some providers as a source of frustration that led to abuse.

*Sometimes providers also lessen their commitment considering the low payment they get compared to their effort. (Midwife #19)*

*Occasionally we hear that, when they get bored or tired, they insult, verbally abuse clients, and throw papers. As they are humans, this could happen when they are tired. I didn’t see this myself though. (Midwifery Student #15)*

Other drivers of abuse consistently identified by both providers and patients were communication difficulties due to the patient and provider not sharing the same language, vocabulary, or concepts, and providers having to care for rural, poorly educated women who might not be familiar with health facilities.

*One* [problem] *is the language barrier when talking with clients from rural places. Midwives do not choose appropriate words for the clients. The clients also could get ashamed when facing the provider. This could be due to the differences in social background the clients came from. (Midwifery Student #1)*

*Sometimes they* [providers] *insult and verbally abuse patients, especially if the patient is from a rural area. It could be because the patient does not understand them well or because the patient is not clean. (Patient #38)*

Several providers also pointed to poor quality care occurring often at high tension junctures in care such as during vaginal exams or when the woman was undergoing labor pains and not able to follow the midwife’s directions:

*Yes I have observed this when the provider wants to do a pelvic examination, the client usually gets afraid to open her legs and the provider shouts and beats her on her leg. (Midwifery Student #14)*

Many also felt that often midwives’ mistreatment of patients was a function of their efforts to provide medically necessary care:

*They do that* [abuse] *for the sake of the mothers. When the labor is in the second stage, and the mother doesn’t care for the baby, the midwives may slap the thigh of the mother only with the aim to save the baby. In this situation, the mother may dislike the midwives, not knowing their* [good] *intention. (Midwife #18)*

We note a discrepancy in patient and provider explanations for abuse. None of the patients mentioned medical necessity or crucial clinical junctures as reasons for mistreatment. Instead they almost exclusively cited provider overwork and lack of cultural competency to care for rural patients as the drivers of abuse.

#### Recommendations for improving quality of care

Recommendations for reducing abuse and improving the quality of care were at best loosely linked to the reasons given for abuse. Midwives’ strongest and most frequently voiced recommendations for improving quality focused on educating communities about the value of midwifery services in preventing maternal mortality. In addition, a strong majority said that they could improve the quality of care they provided if they had better counseling skills to enable them to build a rapport with patients during initial encounters, and to better explain procedures to patients in advance.

*Sometimes the midwife just does what he has to do without creating a good relationship with the client, even without giving the advice she* [the client] *needs. As for the laboring mother, when they are in labor pain, their conduct also changes, and sometimes they may even beat the provider, this also breaks the good relationship. The approach and initial rapport-building phase by the provider are critical. (Midwifery Student #7)*

In several of the midwifery students’ recommendations about improving the quality of care, there is an emphasis on the responsibilities of the patients to speak up for themselves and to communicate better. This at times bordered on placing blame on the patient:

*As they are in pain, mostly the clients do not listen to the midwives when they advise them. She* [the client] *may insult us when we advise her to be positioned on her side. They don’t even listen at all, and sometimes reach to sign for discharge against medical advice.*

***Interviewer*** *: Why do you think that these difficulties occur?*

*They lack the awareness and education. They may even not care for their own baby at this stage; they just want to save their own lives. (Midwifery Student #6)*

*As most clients come from the rural community, they may not be willing to respond to the questions they are asked. They have to be educated. The clients lack education and need to be well counseled to be compliant with what providers tell them to do. (Midwifery Student #10)*

As with providers, patients’ recommendations for improving care also included suggestions for clear, empathetic communication between patients and providers and the need for providers to carry out care in accordance with their training and professional standards. The main thrust of patient recommendations; however, concerned equal treatment of all patients. We found that the more educated patients were keenly aware of the differential in power between midwives and the often uneducated women coming in from rural areas to deliver. They spoke up for these women, and recommended that midwives be especially welcoming to rural patients whom they described as feeling embarrassed and vulnerable during labor.

*Sometimes providers just get upset for the slightest reason. They have to understand that most clients haven’t had the experience of visiting a health facility before. Further, some clients become ashamed or embarrassed when they come to the facility as they have grown up in the rural community. Therefore, the providers should not get upset, given their* [the client’s] *background. (Patient #25)*

Providers also generally recognized the disadvantaged position of their rural patients in accessing services, and some spoke specifically about the special responsibilities of midwives in serving these patients. Both patients and providers emphasized the need for provider “tolerance” and patience with women during labor and delivery.

Patients defined good care as care that was warm, empathetic, and reassuring. The incorporation of traditional birth customs such as providing a coffee ceremony and porridge (*genfo*) to women, which is common in home births, was mentioned by almost all patients as an example of quality care, and frequently cited as a factor in women choosing a health facility for delivery.

*I was aware that they have the coffee ceremony and provide porridge at the Hidassie health center; such practices attract clients and are good. I know a lady who confessed such effect on herself. (Patient #26)*

Although highly valued by patients, provision of customary birth ceremonies was not mentioned by any of the providers or students as an aspect of quality care.

## Discussion

This study examines the experiences of disrespect and abuse in maternal care from the perspective of providers and patients. We find that mistreatment of patients during labor and delivery—particularly verbal abuse—is relatively common and that this abuse has the potential to reduce patient demand for services. Our findings are largely consistent with those from recent international studies of patient mistreatment in maternity services both in terms of the extent of abuse they describe and the triggers for abuse they identify [43, 46]. However, unlike studies conducted in other East African countries, we find no reports of inappropriate demands for payment from midwives, or of detention for non-payment. In addition, reports of abandonment and refusal of care were relatively rare in comparison to other sub-Saharan African studies, and when such abuse was reported, it was not linked to ethnic discrimination or concerns about payment as cited elsewhere [53]. In addition to finding no instances of discrimination based on specific attributes, or of detention in facilities, several themes emerge here that are noted in few other studies, and point to the utility of comparative research on these phenomena between Ethiopia and other contexts.

First, there is an observed discordance between patients and providers in the types of abuse most frequently mentioned. While providers consistently report witnessing physical abuse, patients gave only indirect, anecdotal reports of physical mistreatment. This discrepancy may be in part due to differences in the structure of our interview questions, as providers were asked more directly about abuse than patients. The fact that providers witness more deliveries and spend more time in facilities than patients might also explain differences in reports of physical mistreatment. Finally, although patients seemed open and vocal in their discussions of the quality of care and their experiences of verbal abuse, it could be that they were reluctant to talk about physical abuse, either for fear of retaliation at their local health facility, or because they were uncomfortable discussing traumatic or embarassing events with strangers.

Another area of discordance was the differential patient/provider reporting of abuse stemming indirectly from the poor functioning of the health system rather than the direct actions of providers. Patients frequently reported long wait times, lack of privacy on crowded wards, rushed or abrupt care, and long periods of being left alone during labor as problems that were a form of mistreatment. However, such problems were rarely mentioned by providers as examples of patient mistreatment. Nonetheless, both providers and patients identified “good care” in general as a patient right. It is also notable that both patients and providers did identify sub-standard clinical practices, such as episiotomy without anesthesia or prescription of improper medications, as a type of abuse. Poor clinical care, whether intentional or not, is not a phenomenon that fits neatly in the categories of abuse defined in the Respectful Maternity Care Charter.

Another notable finding here is the role that abuse may play in health-seeking or health-enhancing behaviors. Patients reported that verbal abuse reduced their compliance with instructions, influenced their choice of health facility, and, more importantly, was a factor in their decision-making about whether to give birth at a facility at all, as found elsewhere in Tanzania and Ethiopia [15, 43, 53–57].

A third key finding concerns an area of commonality between patients and providers. Both groups expressed confusion and ambivalence about whether accompaniment and choice of birth positions constitute abuse or a violation of patients’ rights. Patients frequently reported denial of accompaniment and lack of choice in birth positioning, but few identified it as a form of abuse or mistreatment directly. This may reflect a lack of empowerment among patients. Similarly, no providers mentioned these violations of patient autonomy as a form of abuse. This points to an area that may have to be strengthened in future healthcare ethics and patients’ rights training for midwives, as disagreements over positioning can lead to other kinds of abuse.

Our study findings suggest that professional ethics training should be strengthened. We find that ethics and patients’ rights are covered unevenly in the midwifery curriculum. While subjects of privacy and confidentiality are well discussed, issues around respect, patient choice, and autonomy are less thoroughly reviewed. It is also not clear that providers are given the tools to communicate with patients effectively (particularly rural women) or to cope with tense situations where patients resist provider direction. Encouragingly, providers were responsive to additional training on topics of patients’ rights, but mainly because they thought that greater respect for patients’ rights would result in more women coming to health facilities to deliver.

Two final finding of note concern gender and stigma. We find no differences in reports of disrespect and abuse or in knowledge of patients’ rights by gender of the midwife. Moreover, amongst our small sample of patients, we find that male midwives are well accepted, if not preferred, as they were perceived to be more empathetic, in contrast to studies conducted elsewhere [58]. This finding was somewhat unexpected. While reports have indicated that male midwives in Ethiopia are well accepted by patients, it was surprising to find that that male midwives are often seen as being *more* sympathetic and less abusive than female midwives. Our relatively well-educated patient sample may obscure the possibility that rural women with less education might be more receptive to female birth attendants.^3^ Future research on respectful maternity care in Ethiopia should explore gender dynamics in more detail, in particular the relationships between age, patient education levels, perceived lack of provider authority, and gender. For example, it would be interesting to examine whether patients perceive *young* male midwives as having greater legitimacy and authority than their young female counterparts, and whether young female midwives therefore feel a greater need to exert authority in ways that manifest as abuse.

Our study was one of the few to incorporate clinical scenarios involving stigmatized services into an examination of disrespect and abuse during maternity care. The responses to the clinical scenarios indicate that respect for patient autonomy and the right to information and timely care might vary depending on the degree of stigmatization of the service. Further research on the prevalence of disrespect and abuse in stigmatized populations and services would be useful. To date, research on this subject in sub-Saharan Africa has almost exclusively focused on stigma in HIV/AIDS care.

It is important to understand the study results in the context of a health care system that is seeking to expand and improve access to maternity care, but where shortages of staff, facilities, and supplies remain. Our findings suggest that these health system weaknesses are associated with abuse and are seen in themselves as abuse by women. The interviews suggest that abuse most often occurs when harried providers encounter patients who are non-compliant. Patients’ birth stories reveal an undercurrent of provider impatience and haste or rushing to provide care due to over-crowding and heavy workload. Therefore, addressing health systems and structural issues around provider workload should complement any training initiatives on disrespect and abuse of patients. Further operations and evaluation research on the feasibility and effectiveness of these interventions would be required.

Although our findings point to deficiencies in the health system, they also highlight several successes. Notable examples include the high level of knowledge on basic patients’ rights demonstrated by providers, and the absence of reports of corruption and bribery by patients. It is also noteworthy that most of the women interviewed seemed to not be intimidated by health care providers even when abuse occurred (although this might be due to our patient sample being more educated than the national average). They were clear about what they wanted in regards to their care and did not hesitate to point out and criticize lapses. Several patients knew what the obligations of midwives were. As noted above, some also chose facilities based on their reputations for providing quality care, often with the input of health extension workers. This knowledge and strength is a crucial element for building health system accountability, and its presence is an encouraging sign that recent health education and outreach initiatives may have had some success in raising expectations and conveying to women what services they have the right to receive from their local health facilities.

### Limitations

The main study limitations are as follows: our study sample is small and limited to a single geographic region, which makes it difficult to generalize findings to other Ethiopian or African contexts; in addition, we were not able to directly observe the provision of care, so our reports of disrespect and abuse are indirect. The use of clinical scenarios, however, gave us an idea of how providers approach care of patients who might be prone to receiving substandard care and allowed us to gauge whether this care would have involved abuse or an abrogation of rights. Secondly, the use of a single translator limited our ability to conduct a systematic quality assurance of transcript translations.

Further, patients interviewed had a disproportionately high level of education. This is both a limitation and an asset. Educated respondents may be more empowered to speak out about abuse (either experienced or observed) than their less-educated counterparts, so this unusual sample may provide a more accurate picture of the extent of disrespect and abuse among patients than a more representative sample would have. Our less educated patients, however, were far less forthcoming than those reported in other similar studies. This suggests that we may be missing important information on the perspectives of rural, less-educated women in this study. This is a significant limitation, even though we did find that some of our more educated patients seemed inclined to speak up on behalf of those who are least empowered.

## Conclusions

Our findings suggest that policymakers should seriously take recommendations, such as those offered by the participants in this study, to strengthen provider counseling and communication skills. They must also explore ways to structure birth experiences so that they empower women. The study highlights the need for intensive collaboration and dialog between the policymakers, advocates who are concerned with patients’ rights, and those working to improve the quality of RMNCH care, when designing curricula and guidelines for health professional education. They also underscore the very real burdens and constraints faced by midwives in grossly under-resourced health care facilities in these efforts at reform. We hope that they will assist the concerned professional associations to identify priority areas for training as well as for revisions of clinical and supervision guidelines, which have been an increasingly frequent aspect of RMNCH reform efforts [51].

Such work is vital as our findings strongly support the contention that making care at health facilities more women-centered, respectful, and responsive is a crucial component of increasing utilization of maternal health services generally and delivery services in particular, and thus of reducing maternal mortality. It is also fundamental for upholding the rights of women.

## Additional files

Additional file 1: Interview Guides Holcombe & Burrowes v2.pdf. Guide used to interview providers and patients. (PDF 305 kb)

Additional file 2: Codebook Holcombe & Burrowes.pdf. Codebook used for analyzing qualitative data. (PDF 144 kb)

## Abbreviations

DMU: Debre Markos University
RMNCH: Reproductive, Maternal, Newborn and Child Health

## Footnotes

1 While differences in women and men’s cognitive abilities have not been found, there are gender differences in educational access (e.g.*,* enrollment and study time) and achievement expectations in sub-Saharan Africa and elsewhere that can influence test outcomes, as appears to be the case in Ethiopia where males consistently have scored more highly than females in national examinations [37–41]

2 While several institutions, particularly patient support groups (e.g., the International Childbirth Education Association), identify ability to choose preferred birth position as a right (“The Pregnant Patient has the right, after appropriate medical consultation to choose a position for labor and for birth, which is least stressful to her baby and to herself”), there is not international consensus on this point.

3 We are grateful to our reviewers for altering us to this possibility.
