# Supplementary material for: Midwives’ and patients’ perspectives on disrespect and abuse during labor and delivery care in Ethiopia: a qualitative study
Source: BMC Pregnancy Childbirth. 2017 Aug 22;17:263. doi: 10.1186/s12884-017-1442-1 (PMC5567643; doi:10.1186/s12884-017-1442-1)
Supplement: Supplementary file 1 — Interview Guides Holcombe & Burrowes v2.pdf. Guide used to interview providers and patients. (PDF 305 kb) [file 12884_2017_1442_MOESM1_ESM.pdf]

Interview Guide for Practicing Midwives

DEMOGRAPHICS

[Guidance for interviewer: PLEASE START RECORDING IMMEDIATELY AFTER you have received the consent of the respondent and she/he has given consent. For this first section, please write down the answers that the respondents give to these short questions. Please also note the sex of the person - you don't need to ask this question.]

1. Sex **F / M**
2. How old are you? ___________
3. Are you married? **yes / no**
4. Do you have any children? **yes / no**
   a) If yes, how many children have you had? ___________
5. What is your religion? ___________
6. What is your ethnic group? ___________
7. From what region do you originally come? ___________
8. What was your father's level of education? ___________
9. How many years of midwifery training have you had? _____ years
10. How long have you been working as a midwife? ___________
11. At how many deliveries have you been the lead medical professional? _____

[Guidance for interviewer: For the following questions, you do not need to write down what the respondent is saying except for questions 16, 19, 20. We do ask that you note any significant emotions or reactions of the respondent that you observe. For example, please note if an respondent is confused by a question, or if the respondent is upset or uncomfortable answering a particular question.]

12. Why do you think that many Ethiopian women do not deliver with a skilled birth attendant (such as a midwife)?

   ➔ If answer to 12 is training and medical supplies: Other than increasing practical training experience and improving medical supplies are there other reasons that women do not deliver with a skilled birth attendant (such as a midwife)?

13. Again, other than increasing practical training and increasing access to proper medical supplies, what can skilled birth attendants (such as a midwife) do to improve the experience of women who are delivering?

14. Please describe how your training has covered "patients' rights", if at all.

15. What do 'patients' rights' mean to you?

16. What do you believe are the rights of the patient and the responsibilities of the midwife during labor & delivery?
    [Guidance for the interviewer: Please check any mention of the following terms]
    - ☐ Explanation by the midwife of the procedures to be performed
    - ☐ Patient privacy and confidentiality ensured at all times
    - ☐ Respect shown for client/patient privacy
    - ☐ Respect shown to client/patient as an individual
    - ☐ Patient consent obtained for all procedures
    - ☐ Client/patient information kept confidential
    - ☐ Client/patient able to choose her birthing position
    - ☐ Client informed of labor progress
    - ☐ Clients warned of danger signs during Labor & Delivery

17. What difficulties do midwives have in talking with their patients?

18. Why do you think that these difficulties occur?

19. During your practicum or in any facility where you have worked, have you ever observed patients being treated poorly during labor and delivery? **yes / no**

20. If yes, what was the nature of this mistreatment? What happened?

*[Guidance for the interviewer: Do **not** read the items below, but please check any item(s) that the respondent mentions.]*

- ☐ No examples identified
- ☐ **Verbal abuse** (Medical professionals using harsh tones or shouting, using undignified language or threats if mothers don't cooperate)
- ☐ **Physical abuse** (slapping/pinching/hitting, stitching episiotomy without anesthesia)
- ☐ **Procedures conducted without consent** (No explanations on procedures provided to patient)
- ☐ **Confidentiality violated** (medical results shared when others could hear)
- ☐ **Mother's right to privacy not respected** (mother not covered when moved to delivery room or not covered after delivery)
- ☐ **Medically unjustified and culturally insensitive restrictions on the mother** (such as denying drink and food during labor, denying liberty of movement during labor, denying choice of position for delivery)
- ☐ **Discrimination** based on mother's age, marital status, ethnicity, race or economic status (such as worse treatment of younger or unmarried mothers, detention of the woman in facility due to lack of payment of facility fees)
- ☐ **Performing harmful practices** (such as excess of vaginal examination, unnecessary separation of mother and newborn after the delivery, poor infection prevention practices, etc.)

- ☐ **Abandonment of care of mother (**mother left unattended, mother ignored while asking for pain relief/medication)
- ☐ **Inappropriate use or overuse of drugs and technology** (like **episiotomy**, etc.)
- ☐ **Other** (specify) ____

Care scenarios *(Interviewer: Please read each scenario to the respondent and then ask the two questions below it.)*

I am going to read you three different scenarios about patients seeking care from a midwife. After reading each scenario, I will ask you a general question about what you would do in the situation. I will next ask about how confident you would be caring for this patient and why.

Woyzero Yeshi, a 34 year-old woman, came to the health post at 16:00 hrs. A traditional birth attendant (TBA) had earlier delivered W/o Yeshi's healthy baby girl at home at 04:00 hrs. W/o Yeshi's family brought her to the clinic because they were concerned because she seemed to be bleeding excessively. When she arrived, the nurse estimated that she had lost at least 350 ml of blood. W/o Yeshi's pulse was 95 and her blood pressure was 105/60.

21. What would you do?

22. How confident would you feel in caring for this patient?

Very Confident **Confident** **Neutral** **Not Confident** **Not Confident at all**

- Why?

Woyzerit Miriam, an unmarried 14 year-old patient, comes in to your clinic. She works as a house maid in the town where this health facility is located. She asks for a contraceptive method. She also asks that you not tell her parents or other relatives or employers.

23. What would you do?

24. How confident would you feel in caring for this patient?

Very Confident **Confident** **Neutral** **Not Confident** **Not Confident at all**

- Why?

Woyzero Tsehai, a 24 year-old married woman with three children, comes into your health post and requests that you help her with an abortion. The health post is 8 hours away from any other health facility,

25. What would you do?

26. How confident would you feel in caring for this patient?

Very Confident **Confident** **Neutral** **Not Confident** **Not Confident at all**

- Why?

Is there anything more that you want to tell us that will help us better understand any difficulties in the interactions between patients and providers that could affect the quality of care patients receive?

Thank you for your time and responses. I now have one page with a few questions for you to check off.

Separate Questionnaire Sheet for Midwives and Midwifery Students

A. Which of the following do **you** believe are the rights of the patient during labor & delivery? Please check (Y/N).

Explanation by the midwife of the procedures to be performed (Y/N)
Patient privacy and confidentiality ensured at all times (Y/N)
Respect shown for client/patient privacy (Y/N)
Respect shown to client/patient as an individual (Y/N)
Patient consent obtained for all procedures (Y/N)
Client/patient information kept confidential (Y/N)
Client/patient able to choose her birthing position (Y/N)
Client informed of labor progress (Y/N)
Clients warned of danger signs during Labor & Delivery (Y/N)

B. Were any of the above items covered in your training? **Yes/No**

C. In your work or clinical training, have you ever encountered any of the following types of behavior by medical professionals? Please check (Yes/No) the behaviors that you have observed on the list below.

No examples identified (Y/N)
Verbal abuse (Medical professionals using harsh tones or shouting, using undignified language or threats if mothers don't cooperate) (Y/N)
Physical abuse (slapping/pinching/hitting, stitching episiotomy without anesthesia)
Procedures conducted without consent (No explanations on procedures provided to patient) (Y/N)
Confidentiality violated (medical results shared when others could hear) (Y/N)
Mother's right to privacy not respected (mother not covered when moved to delivery room or not covered after delivery) (Y/N)
Medically unjustified and culturally insensitive restrictions on the mother (such as denying drink and food during labor, denying liberty of movement during labor, denying choice of position for delivery) (Y/N)
Discrimination based on mother's age, marital status, ethnicity, race or economic status (such as worse treatment of younger or unmarried mothers, detention of the woman in facility due to lack of payment of facility fees) (Y/N)
Performing harmful practices (such as excess of vaginal examination, unnecessary separation of mother and newborn after the delivery, poor infection prevention practices, etc.) (Y/N)
Abandonment of care of mother (mother left unattended, mother ignored while asking for pain relief/medication) (Y/N)
Inappropriate use or overuse of drugs and technology (like **episiotomy**, etc.)
Other (specify) ____

Thank you for your time and contribution to strengthening the quality of midwifery.

Discussion guide for women who have given birth at a health center <u>with a midwife</u>

[<u>Guidance for interviewer</u>: <u>PLEASE START RECORDING IMMEDIATELY AFTER</u> you have received the consent of the interviewee. Please read the following statement.]

We would like to learn about the experience women have when they receive pregnancy care. Thank you for agreeing to help us learn about this topic.

[<u>Guidance for interviewer</u>: For this first section, please write down the answers that the interviewees give to these short questions.]

Demographic information

1. How old are you?
2. What is your marital status? ___________
3. What is your religion? ___________
4. What is your ethnic group? ___________
5. How many years of schooling have you had? ___________
6. How many children have you had? ___________
7. Was your last delivery in a health facility? **yes / no**
8. Where do you live? ___________
9. How long does it take to walk from your house to your health center? ___________

10. Were you assisted by a midwife for your most recent pregnancy and/or delivery? **yes / no**

[<u>Guidance for interviewer</u>: If no, skip to question 19)

11. Was your midwife male or female? **F / M**

12. If you had the choice, would you prefer to be seen by: (*Read out the options*)
    A female provider   A male provider   No preference

13. What kinds of services did you get from the midwife?
    - *Probes: health education, care during labor and delivery, post-partum care, breastfeeding support*

14. How do you generally feel about the services that you got from the midwife? *Probes: Would you please explain how and why? What do you like, what do you not like (like less)?*

15. When you came to the health center to get care, how did you feel about talking to your midwife?
    - Did you feel that you could ask questions? **yes / no**
    - Was there a question that you wanted to ask the midwife or other medical professional but felt uncomfortable asking?

16. Do you think that midwives understood and were sensitive to your traditional beliefs and practices when you came to the health center for care related to pregnancy? (*prompts: were you allowed to have a family member accompany you? Were you allowed to give birth in a position you wanted? Were you allowed to eat and drink during labor?*)

17. Would you have liked the midwives to do anything different when you came in for pregnancy-related care? **yes / no**

[Guidance for interviewer: Skip to question 19 if woman answers 'No' to question 17.]

18. If yes, what would you like the midwife to have done differently?

19. Were you satisfied with the services you received? **yes / no**

20. Have you ever heard about a pregnant woman from your community who has had a problem with the care from a midwife or other similar medical professional at the health center? **yes / no**

[Guidance for the interviewer: If no to question 20, skip to question 22

21. If yes, what was the nature of the problem, what happened?

[Guidance for the interviewer: Please check each item below that the respondent mentions.]

- ☐ neglect or lack of attention from the midwife or other similar medical professional
- ☐ verbal abuse from the midwife or other similar medical professional?
- ☐ physical abuse from the midwife or other similar medical professional?

22. What suggestions could you make that would help midwives do a better job of helping women who are delivering?

23. What suggestions could you make that could improve the communications between patients and midwives?

24. Is there anything else that you think we should know about the kind of care pregnant women in the community get from midwives at the health center?

Thank you for your time and responses and contribution to strengthening the quality of midwifery.
