# Supplementary material for: Midwives’ and patients’ perspectives on disrespect and abuse during labor and delivery care in Ethiopia: a qualitative study
Source: BMC Pregnancy Childbirth. 2017 Aug 22;17:263. doi: 10.1186/s12884-017-1442-1 (PMC5567643; doi:10.1186/s12884-017-1442-1)
Supplement: Supplementary file 2 — Codebook Holcombe & Burrowes.pdf. Codebook used for analyzing qualitative data. (PDF 144 kb) [file 12884_2017_1442_MOESM2_ESM.pdf]

TOURO UNIVERSITY, CALIFORNIA - DEBRE MARKOS UNIVERSITY WOMEN-CENTERED MIDWIFERY PILOT STUDY CODEBOOK

Midwifery Student/Midwife Demographic Data

Interview number, Sex; Education; Marital status; Parity (birth); Age; MW type (Bachelor's Degree or Accelerated Midwife; practicing or student); Facility work at (for midwives)

Table 1: Midwifery Students and Midwives Codebook

CODE	EXPLANATION	NOTES
MIDWIVES' TRAINING		
Satisfaction with training	Whether happy with training	
Adequacy of training	Sufficiency of training (practical, didactic), whether there are any perceived gaps<br><br>Subcodes (list of specific issues mentioned)<br>• Equipment<br>• Practice time<br>• Variety of experience	
Coverage of professional ethics in training	Does the respondent indicate whether this topic was covered during training, if yes, how does she describe it?<br><br>Subcodes (list of specific rights mentioned)<br>• Privacy<br>• Confidentiality	
Coverage of patients' rights in training	Does the respondent indicate whether this topic was covered during training, if yes, how does she describe it?<br><br>Subcodes (list of specific rights mentioned)<br>• Privacy<br>• Confidentiality	
Respondent's knowledge and understanding of patients' rights	Which elements of patients' rights the respondent identifies during the interview conversation (whether leaves anything out on the survey form)	
PERCEPTIONS OF FACILITY-BASED DELIVERY		
Views on rationales for client use/non-use of facility-based labor and delivery services	How midwife understands why some women do not come to deliver at health facilities; his/her assessment of the validity of these rationales<br>Subcodes (list of specific rationales mentioned)<br>• Language<br>• Ethnicity<br>• Culture<br>• Education<br>• Distance<br>• Poor facility quality	
Social and traditional beliefs and practices and facility-based labor and delivery services		
Recommendations as to how to improve treatment of clients		
Recommendations as to how to improve communication with clients		
NATURE OF CLIENT-MIDWIFE INTERACTIONS (types of violations of rights/abuse observed)		
Midwives and privacy during labor and delivery	Whether and how the midwife describes any violations of privacy during care she/he has provided, or that she/he has	

CODE	EXPLANATION	NOTES
	observed, examples if any (e.g., leaving the client uncovered, conducting vaginal exams when others are around, etc.) Whether or not she/he recognizes these as violations of privacy	
Client-Midwife communications		
Midwives and confidentiality during labor and delivery	Whether and how the midwife describes any violations of confidentiality by herself or other midwives, whether or not she recognizes these as a violation of confidentiality (e.g., talking about the client's case when other patients can hear)	
Midwives and insults	Whether the respondent midwife has insulted/berated a client, or whether the respondent has heard another midwife berate clients	
Positioning for birth	Whether the respondent allows the client to choose her own position for delivery	
Presence of relatives during labor and delivery (if desired)	Whether client's relatives were present at labor and delivery; whether she requested this; whether she wanted this	
Medical errors	Recognition of whether medical errors are made during the course of the care the midwife provided or observed being provided	
Explanations for violations of rights observed	Subcodes<br>• Adequacy of equipment in the health facility<br>• High patient load<br>• Stress<br>• Culture/Traditional practices<br>• Language/communication barrier<br>• Unresponsive patient<br>• Education	

Criteria for Coding Clinical Care Scenarios

Post-partum hemorrhage

- Did they make the correct clinical recommendations? (completely; majority)

Unmarried adolescent seeking contraception

- Did they refuse care? [Code: Was not willing to provide care]
- Did they require parental consent? [Code: Directive counseling or unnecessary procedures]
- Did they tell the patient what they should do rather than finding out what they wanted? [Code: Directive counseling or unnecessary procedures]

Married adult seeking a medical abortion

- Did they refuse care? [Code: Was not willing to provide care]
- Did they insult the client [Code: Displayed stigmatizing attitudes]
- Do they try to validate why the client is asking for an abortion (other than legal eligibility) [Code: Directive counseling or unnecessary procedures]
- Did they know what the law is (e.g., in what situations abortion is permitted and what the woman needs to demonstrate – nothing)?
- Did they require partner consent? [Code: Directive counseling or unnecessary procedures]

Client Demographic Data

Interview number; Sex; Marital status; Education; Parity (birth); Age; Facility where gave birth

Table 1: Client Codebook

CODE	EXPLANATION
PERCEPTIONS OF FACILITY-BASED DELIVERY	
GROUP: Explanations for client use or non-use of facility-based labor and delivery services	How client understands why some women do not come to deliver at health facilities; their assessment of the validity of these rationales
Reason for nonuse: Language barriers	Specific rationales mentioned
Reason for nonuse: Culture	
Reason for nonuse: Lack of education	
Reason for nonuse: Distance to facility	
Reason for nonuse: Poor facility quality	
PERCEPTIONS OF MIDWIVES	
GROUP: Perceptions of midwives' quality of services	Respondent's assessment of how good or bad the care that midwives provide is generally<br>*(use mistreatment codes for reasons for negative perceptions of midwives)*
Positive perception midwives	
Negative perception midwives	
Reason positive: Reassuring midwife	
Reason positive: Knowledgeable/competent midwife	
Reason positive: Friendly/relaxed midwife	
Reason positive: Coffee ceremony	
Reason positive: Quick labor	
Reason positive: Food and drink provided	
Reason positive: Easy labor (lack of pain, complications, etc)	
Reason negative: Complicated labor, poor outcome	
GROUP: Perceptions of male vs. female midwives	Whether the client expresses any preference – or none at all – about midwives
Prefer male midwife	
Prefer female midwife	
No preference male/female midwife	
Reason prefer male: competence, professionalism	
Reason prefer male: greater empathy	
Reason prefer female: modesty	
Reason prefer female: familiarity	
OWN & OBSERVED EXPERIENCE OF LABOR AND DELIVERY	
GROUP: Personal experience of care during labor & delivery	Client satisfaction with their care in L&D<br><br>*Use "**reason positive**" midwifery codes to code conversation about birth experience, and abuse/quality codes to code conversation about negative experience*
Personal experience L&D: good	
Personal experience L&D: bad	
Personal experience L&D: mediocre	
GROUP: Context of abuse	Whether the respondent is discussing own experience, observed experience or what have heard others experience
Own abuse discussed	
Observed abuse discussed	
Anecdotal abuse discussed	
No abuse found/discussed	
GROUP: Abuse type	
Interpersonal mistreatment	
Service delivery mistreatment	
GROUP: Violations of *privacy* during labor and delivery	Whether and how the respondent describes any violations of privacy during her delivery
Abuse privacy: Patient left uncovered	
Abuse privacy: Public vaginal exams	
Confidentiality violated during labor and delivery	Whether the respondent describes any violations of confidentiality during her delivery

CODE	EXPLANATION
GROUP: Verbal Abuse	Whether the midwife has insulted/berated the respondent, or whether the respondent has heard the midwife berate other patients
Verbal abuse: insults/rudeness	
Verbal abuse: shouting/yelling/harsh tones	
Verbal abuse: mocking/dismal	
GROUP: Positioning for birth	Respondent experience of bodily position for childbirth; whether or not she was allowed to choose her preferred position.
Allowed to choose birthing position	
Not allowed to choose birthing position	
Not allowed to chose birthing position: health of baby cited	
GROUP: Presence of *relatives* during labor and delivery (if desired)	Whether respondent's relatives were present at labor and delivery; whether she requested this; whether she wanted this
No accompaniment: did not want	
No accompaniment: did not want: privacy concerns	
No accompaniment: did not want: do not want to worry relatives	
No accompaniment: wanted but not allowed	
GROUP: Poor clinical practice	Whether the respondent mentioned poor clinical practice during the course of the care
Poor clinical practice: improper medication	
Poor clinical practice: denial of pain medication, procedures without anesthesia	
Poor clinical practice: abandonment	
Poor clinical practice: unnecessary procedures	
Poor clinical practice: slow service, long wait	
GROUP: Other Abuse	
Lack of communication about procedures being performed	Whether the midwife communicated about procedures that were to be performed
Physical abuse: Hitting/slapping/pinching of patients	
GROUP: Explanations for violations of rights observed/experienced	
Explanation abuse: inadequacy of equipment in the health facility	
Explanation abuse: Poorly educated patients	
Explanation abuse: High patient load, heavy workload	
Explanation abuse: Stress	
Explanation abuse: Language/communication barrier	
Explanation abuse: Uncooperative patient	
Explanation abuse: Lack of patient education	
GROUP: Suggestions for improving practice	
To improve: make interactions more fun and lighthearted	
To improve: prioritization of high risk patients	

Features of interviews with women/patients/clients

Responses ever more than one sentence? Questions understood or not? Ability to ask questions during interview?
